# Eating or Meeting? Cluster Analysis Reveals Intricacies of White Shark (*Carcharodon carcharias*) Migration and Offshore Behavior

**DOI:** 10.1371/journal.pone.0047819

**Published:** 2012-10-29

**Authors:** Salvador J. Jorgensen, Natalie S. Arnoldi, Ethan E. Estess, Taylor K. Chapple, Martin Rückert, Scot D. Anderson, Barbara A. Block

**Affiliations:** 1 Monterey Bay Aquarium, Monterey, California, United States of America; 2 Hopkins Marine Station, Stanford University, Pacific Grove, California, United States of America; 3 Université Paris VI, Paris, France; 4 Point Reyes National Seashore, Inverness, California, United States of America; University of Canterbury, New Zealand

## Abstract

Elucidating how mobile ocean predators utilize the pelagic environment is vital to understanding the dynamics of oceanic species and ecosystems. Pop-up archival transmitting (PAT) tags have emerged as an important tool to describe animal migrations in oceanic environments where direct observation is not feasible. Available PAT tag data, however, are for the most part limited to geographic position, swimming depth and environmental temperature, making effective behavioral observation challenging. However, novel analysis approaches have the potential to extend the interpretive power of these limited observations. Here we developed an approach based on clustering analysis of PAT daily time-at-depth histogram records to distinguish behavioral modes in white sharks (*Carcharodon carcharias*). We found four dominant and distinctive behavioral clusters matching previously described behavioral patterns, including two distinctive offshore diving modes. Once validated, we mapped behavior mode occurrence in space and time. Our results demonstrate spatial, temporal and sex-based structure in the diving behavior of white sharks in the northeastern Pacific previously unrecognized including behavioral and migratory patterns resembling those of species with lek mating systems. We discuss our findings, in combination with available life history and environmental data, and propose specific testable hypotheses to distinguish between mating and foraging in northeastern Pacific white sharks that can provide a framework for future work. Our methodology can be applied to similar datasets from other species to further define behaviors during unobservable phases.

## Introduction

Highly migratory apex marine predators such as tunas and sharks likely impose ecological pressures across a variety of different ecosystems including coastal and open ocean habitats [1]. Such broad movement prohibits continuous direct observation of fine-scale behaviors and ecological interactions. Pop-up satellite archival transmitting (PAT) tags have been developed to describe these unobservable phases. However, technological and cost restrictions have for the most part limited PAT tags to three sensors which archive light, pressure and temperature observations, used to determine geographic position, swimming depth and environmental temperature, respectively. Though these parameters are instrumental in describing core areas and environmental preferences, differentiating the functional significance of habitat use has remained challenging beyond broadly categorizing behaviors such as ‘transiting’ and ‘foraging’ based upon observations of more linear and more tortuous horizontal movement patterns [2]. There remains a strong need for novel analysis methods to provide information on finer scale behaviors within these broad categories, to better understand how mobile ocean predators utilize the ecosystems they inhabit.

The oceanic migrations of white sharks (*Carcharodon carcharias*) have only recently been described following the advent of PAT tags [3]–[8]. From these tagging studies the seasonal patterns of long-distance migrations have emerged in the northeastern Pacific. Mature white sharks, and some sub-adults (adolescents), spend a portion of time around coastal aggregation sites (approximately August to January), primarily near pinniped rookeries, and another portion offshore in pelagic habitats. The offshore habitat extends from the western coast of North America, between Mexico and Canada, to the Hawaiian Archipelago as far west as Midway Island. White sharks range throughout this pelagic habitat, but much of the activity, particularly for males, is concentrated in a region centered approximately halfway between the Baja peninsula and the big island of Hawaii [8]. This area has been referred to as an offshore focal area [6], a shared offshore foraging area [7], and the ‘white shark Café’ [8].

Animals undertake extensive migrations for a number of reasons including escape, dispersal, foraging and reproduction [9]. The seasonal migratory movements of white sharks from coastal California to offshore waters was first observed by Boustany et al. [3] who hypothesized it was most likely related to some type of foraging or reproductive opportunity. Weng et al. [6] noted that one tagged white shark of unknown sex made rapid vertical movements in the water column for extended period while offshore, and suggested a potential courtship behavior. Domeier and Nasby Lucas [7] noted that white sharks tagged off Guadalupe Island, Mexico, did not visit California but visited the same offshore locations including the Café - the primary area where the two groups overlapped seasonally. They concluded that this must be a common foraging area, given a large geographical expanse, and apparent sexual separation (also see [10]). Jorgensen et al. [8] noted the Café was primarily defined by the presence of males converging during spring within a much smaller core area coincident with an increased rate of vertical movement while females visited the Café center only briefly. They cited this as support for a potential mating area. Recent empirical evidence from stable isotopic analysis of white shark tissues sampled off the coast of California confirmed that white sharks foraged while offshore, but questioned whether foraging was the primary benefit, since the rate of prey consumption offshore was estimated to be approximately half of that occurring at the coast [11]. Unfortunately, difficulty in observing sharks during this offshore phase and limitations in PAT data analyses have made behavioral and physiological conclusions elusive. While foraging or mating remain the primary candidate explanations, specific hypotheses that can be tested with available or foreseeable methods are needed to ultimately elucidate this important aspect of white shark ecology specifically in this offshore location.

Patterns of vertical behavior recorded from electronic tags can be an effective tool to infer behavior in fishes and sharks [12]–[15] such as courtship/spawning behavior [16], [17]. A number of methods have been effectively used to analyze detailed fine-scale archival depth data to differentiate behaviors (reviewed in Bradford et al. [18]). Due to behavioral variability among individuals it is important to have large sample sizes to attain generalizable results, which has been a limiting factor. Large PAT tag datasets exist for white sharks, however, current analyses of vertical behavior have relied on the use of archival data from rarely recovered PAT tags, which are rich in detail but poor in general representation due to low replication of individuals [5]–[8], [19]–[21].

To date, some specific patterns in white shark vertical behavior have been identified, along with hypotheses about their ecological meaning. Goldman and Anderson [22] identified signature swimming depth patterns in white sharks patrolling near seal rookeries at the Farallon Islands from active tracking (primarily between 30 m and just below the surface). Boustany et al. [3] showed this coastal signature changed as individuals left the coast and began migrating, at which point they swam primarily at the surface with infrequent dives to 500 m. Weng et al. [6] showed that while offshore, white sharks engaged in ‘rapid oscillatory diving’ (ROD), noting that one individual made repeated vertical excursions below the surface mixed layer up to 96 times in 24 hours. A subsequent study demonstrated that ROD occurred primarily in the Café as males converge there during spring [8]. A fourth pattern, identified from individuals near Hawaii closely mirrored the diel vertical migration typical of the deep scattering layer (DSL) [23] and was attributed to foraging within that community [8].

An important methodological objective of the present study was to provide a new dive behavior analysis approach using entire PAT tagging datasets, not just recovered tags with detailed archived data, to ascertain more behavioral information than previously possible. To accomplish this we used a previously published *C. carcharias* PAT tag dataset [8] and applied a clustering analysis to transmitted summary records to objectively differentiate dive behavior modes throughout the population's range in the northeastern Pacific. We then sought to use the subset of records with full archival data to validate the results. Finally, we looked for spatial, temporal and sex-based patterns in the diving behavior to further inform the discussion of the potential for foraging and/or mating in the white shark Café.

## Materials and Methods

### Ethics Statement

This project was conducted with permits from the California Department of Fish and Game, National Oceanographic and Atmospheric Administration, Office of the National Marine Sanctuaries (under permit MULTI-2005-005; MULTI-2009-005), U. S. National Park Service, and under Stanford University animal care protocol 10765 which specifically approved the tagging methodologies used in this study.

White sharks were tagged with PAT tags in central California as previously described in Jorgensen et al. [8]. A table detailing the deployments of all tags analyzed in this study is available in the online data supplement at http://rspb.royalsocietypublishing.org/content/277/1682/679/suppl/DC1.

Time-at-depth histogram data were compiled from transmitted PAT data. Tags were programmed to record and transmit histogram data for each 24-hour period into depth range bins. We used 11 bins with edges defined by 0, 5, 10, 50, 100, 150, 200, 250, 300, 500, 700, and 1000 m. Depth records <0 m were assumed to be zero. In addition to 44 transmitted records, we incorporated archival records from nine recovered PAT tags. We recreated equivalent binned data using a simple script created with Matlab (Mathworks, v 7.9.529) and compiled these with the transmitted records for a total of 53 individual tag records with between 7 and 361 (mean = 105) days of histogram data and a total of 5571 days. Of the 53 individuals 25 were male, 20 were female and eight were of unknown sex.

We then calculated a distance matrix to determine the similarity among the 5571 days based on differences in vertical distribution in the 11 depth bins (‘pdist’ function in Matlab, Mathworks, v 7.9.529). For distance calculations we used the ‘City block’ measure, also known as the ‘Manhattan’ measure. The ‘Euclidian’ distance measure (geometric straight line) is likely the most common distance measure for clustering analysis. However, since distance is computed in multi-dimensional space, the ‘Euclidian’ measure is inappropriate if dimensions are of differing scales. In this case, since the bin data were of varying depth ranges (from 5 to 300 m), the method would tend to be biased by those dimensions with larger scales. Instead we calculated ‘City block’ distance, also a very common measure, which is simply the average distance along each dimension, and is more appropriate for discrete data. To maximize contrast between days we set bin values <0.1 equal to zero, essentially de-emphasizing potential outliers and rare events.

From the distance matrix, we created a hierarchical cluster tree using an un-weighted average distance (UPGMA) linkage algorithm (‘linkage’ function in Matlab, Mathworks, v 7.9.529). These resulting clusters were plotted along with a dendrogram using the Matlab ‘dendrogram’ function.

For each day represented by depth histogram data, a median geographic position (latitude and longitude) was estimated by fitting geolocation data to a Bayesian state space model according to previously described methods [24]. These positions were grouped by cluster and plotted to detect geographic, seasonal and sex based behavior patterns.

Archival data in 60 sec resolution were available for nine individuals, and comprised 43% of available histogram days. Following the clustering procedure, the days with archival data became interspersed among the clusters. These data were queried by cluster to examine differences in diving patterns. This step was to investigate diel patterns not discernible from 24-hour histograms, and trends in vertical velocities determined from the change in measured depth between successive (60 sec) readings. To visualize diel patterns we compiled all archival data for each cluster and plotted the density of depth readings over a single 24-hour cycle. Because the range of the white sharks spanned 60° of Longitude, we aligned the data to correspond to local time for the 24 h plots. This was done by interpolating the longitude estimate for each datum along the estimated track using the Matlab function ‘interp1’ with the ‘spline’ method, and then adjusting for local time according to longitude.

To determine the spatial dependence of specific behavior modes in the Café, we calculated the proportion of data from each mode versus their geographic position within the Café. This analysis was done using longitude, since longitude estimation is more precise and latitude estimation can be confounded by non-normal spatially dependent error [24]. The Café center was defined by the local maximum in the distribution of longitude estimates across the entire data set. We used simple linear regression to examine the prevalence of different behavior modes as a function of distance from the Café center.

To determine the fraction of time male and female white sharks engaged in particular behaviors while in the Café (±10 degrees of longitude from the Café center) we calculated the fraction of days each individual engaged in each behavior for each month. We then compared the median of individuals (for males and females) by month.

## Results

**Figure 1:**
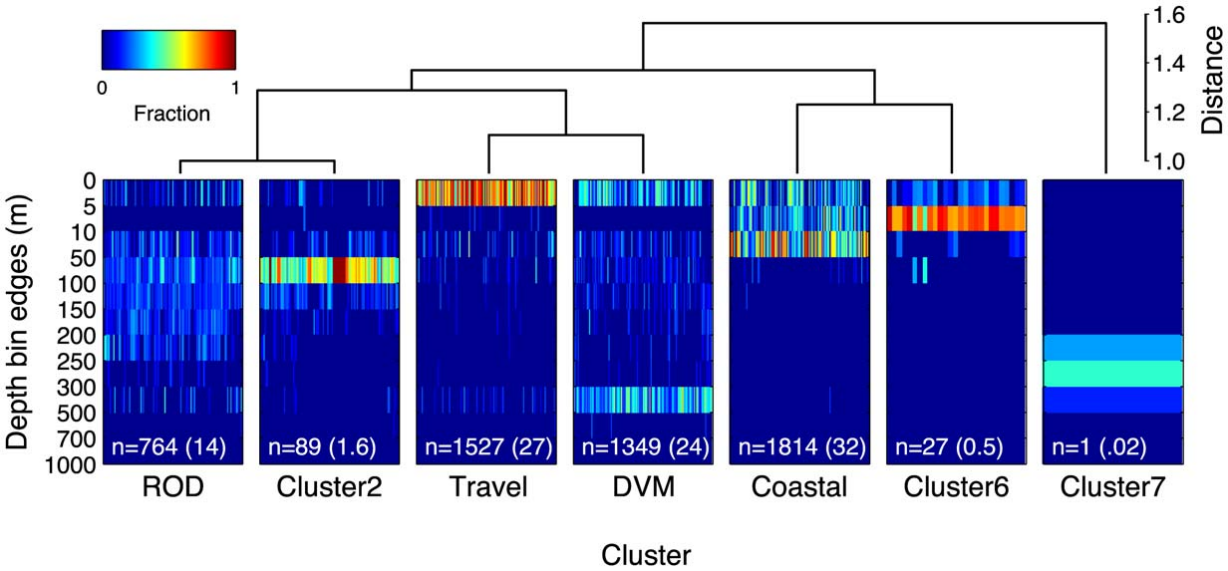
Dendrogram of white shark behavior, determined from clustering analysis of differences in diving patterns. Each column represents a 24-hour depth histogram (n = 5571 days from 53 sharks) and is colored by fraction of time. Distinct vertical distribution patterning is evident in the grouping of days with similar depth distributions. The size of each cluster, is indicated by the number of days (n), and percent of total days (in parentheses). The density variable is expressed as a fraction of each day spent in depth bins defined along the y-axis.

Four groups emerged as the dominant diving behavior modes from the cluster analysis. These four groups accounted for over 97.9% of the data. Three additional groups were differentiated (clusters 2, 6 and 7), however, they represented only 1.59, 0.48, and 0.02% of the data respectively (Figure 1). The dendrogram plot (Figure 1) illustrates the relative distance between groups, representing differences in diving behavior. These clear differences are evident from the depth-bin histogram plots for each cluster.

**Figure 2:**
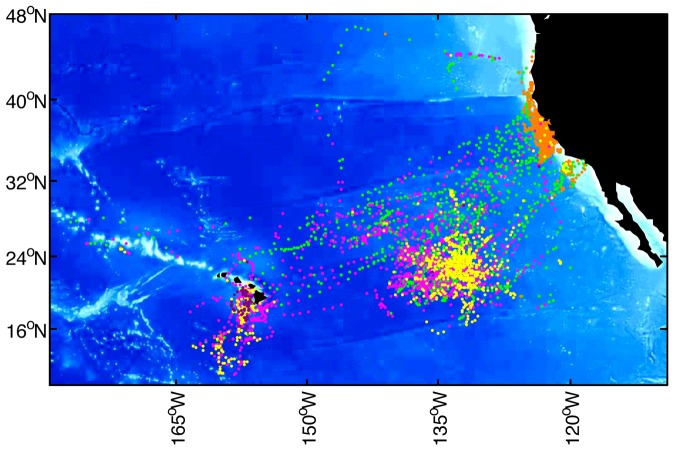
Daily median white shark position estimates from 53 tracks, Each position estimate is colored according to behavioral cluster; cluster 1 (yellow; ‘ROD’), cluster 2 (purple; ‘Cluster 2’), cluster 3 (green; ‘Travel’), cluster 4 (magenta; ‘DVM’), cluster 5 (orange; ‘Coastal’). The distinct diving behaviors, distinguished by each cluster, generally differed in the locations where they most commonly occurred. The ‘Coastal’ behavior occurred primarily along the North American coast, ‘ROD’ primarily at the ‘white shark Café’, while ‘DVM’ occurred throughout the offshore area (the Café, Hawaii, and in between) and ‘Travel’ connected North America and the offshore core areas (the Café and Hawaii).

The distinct diving modes showed spatial patterning across the white sharks' migratory range (Figure 2). Cluster 5 (orange; ‘Coast’) grouped along the west coast of North America, cluster 3 (green; ‘Travel’) occurred throughout the range, but was dominant between the coast and the offshore habitat, particularly in between ‘core areas’, presumably along the migratory route. Cluster 4 (magenta; ‘DVM’) occurred throughout the offshore area but was most concentrated in the Café and near Hawaii. Cluster 1 (yellow; ‘ROD’) was most prevalent in the Café area, with some occurrence near Hawaii. Cluster 2 (purple) was confined to a small area south of Hawaii, and only comprised 1.59% of the data (see Figure 1).

**Figure 3:**
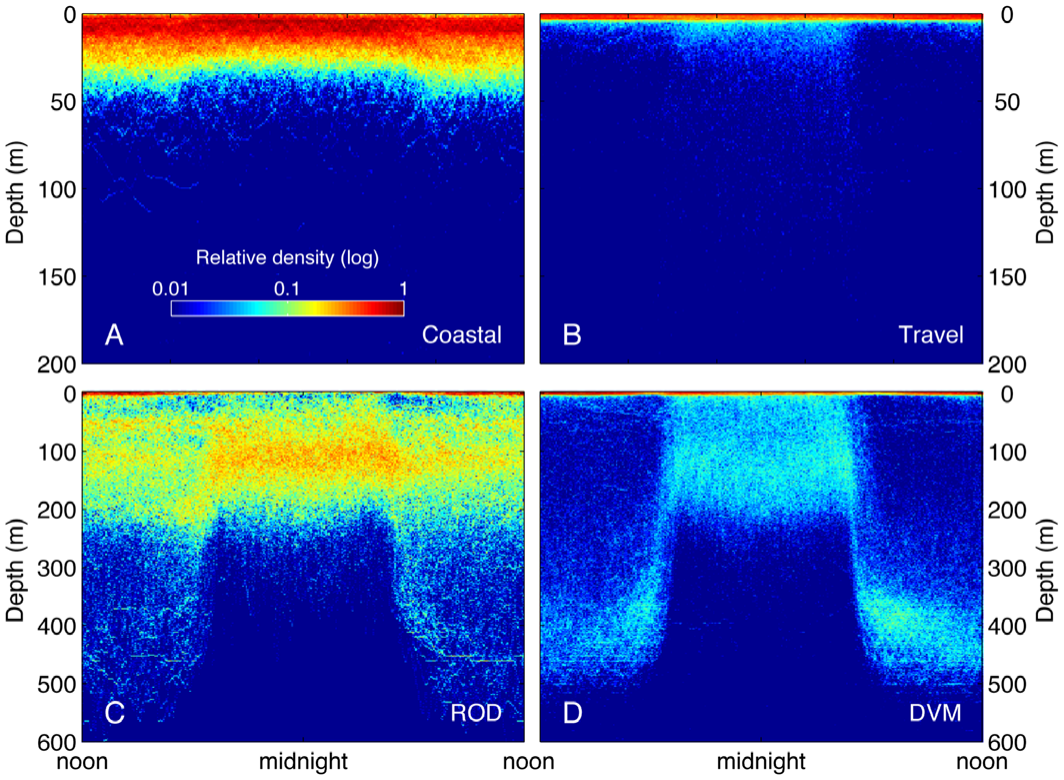
Diel patterns of white shark diving behavior from archival records queried by cluster. High-resolution time and depth data were corrected for local time and aggregated by cluster over a 24-hour period. The relative density of data (log scale) is shown on a gridded surface for the four dominant clusters ‘Coastal’ (A), ‘Travel’ (B), ‘ROD’ (C), and ‘DVM’ (D) representing 97.9% of data. The clusters differed not only by overall daily depth distribution, but also in the diel pattern of depth occupancy. Note that a faint signal of DVM dive pattern can be detected in the other clusters. This is likely because sharks switched between behaviors at arbitrary times of day while the data were divided in regular 24-hour bins, resulting in some ‘bleeding’ of the behavior across clusters.

To better understand the vertical behavior representing each diving mode, we plotted depth versus time of day using the subset of archival data (from recovered tags) queried for each cluster. These plots revealed in more detail the dominant depths used, and distinctive diel patterning of the diving behaviors (Figure 3). Since cluster 5 occurred almost exclusively along the coast of North America (Figure 2), we refer to it as the ‘Coastal’ behavior mode. The ‘Coastal’ mode was characterized by vertical distribution shallower than 50 m and mostly in the upper 30 m of the water column. Day/night differences were subtle (Figure 3a). Cluster 3 was characterized by swimming at the surface with only faint traces of deeper activity (Figure 3b). We refer to cluster 3 as the ‘Travel’ mode since this behavior occurred primarily along the connecting corridors between the coast and the Café, and Hawaii, respectively, and because it matches a well-described pattern of white shark vertical behavior (mainly surface swimming) characteristic of uninterrupted travel [4], [6], [7], [25].

Cluster 1 was distinguished by a relatively even distribution between 30 and 200 m. During night this was somewhat more concentrated near 100 m (+−50 m) and during daylight there was also a substantial fraction of time spent at the surface. We refer to cluster 1 as the ROD mode (Figure 3c), thus identified by the large increase in vertical velocity compared with all other modes (Table 1). This rapid diving pattern resulted from repeated up and down vertical movements between the approximate depths of 30 and 200 m for periods extending over days [6], [8].

**Table 1:** White shark mean vertical velocities for different dive behavior clusters measured from a subset of archival records from recovered PAT tags.

Mean Vert. vel.	Coastal	Travel	DVM	ROD	ROD HI	ROD Café
All* (m/s)	0.043	0.072	0.197	0.430	0.107	0.557
Male (m/s)	0.047	0.093	0.291	0.572	N/A	0.573
Female (m/s)	0.041	0.048	0.093	0.085	0.085	0.106
N (All individuals)	9	9	9	9	4	5
N (All days)	692	681	586	346	84	250

* _All_ includes male, female and unknown sex.

Cluster 4 showed strong differences between day and night vertical distribution (Figure 3d) with a daytime peak centered between 350 and 500 m, a nighttime peak in the upper 200 m, and a clear dusk and dawn vertical migration between depths. We refer to this cluster as the DVM mode; a diel vertical migration pattern previously described for white sharks and other oceanic species [8], [15]. Additionally, in this cluster, a substantial amount of time was spent swimming at the surface day and night.

**Figure 4:**
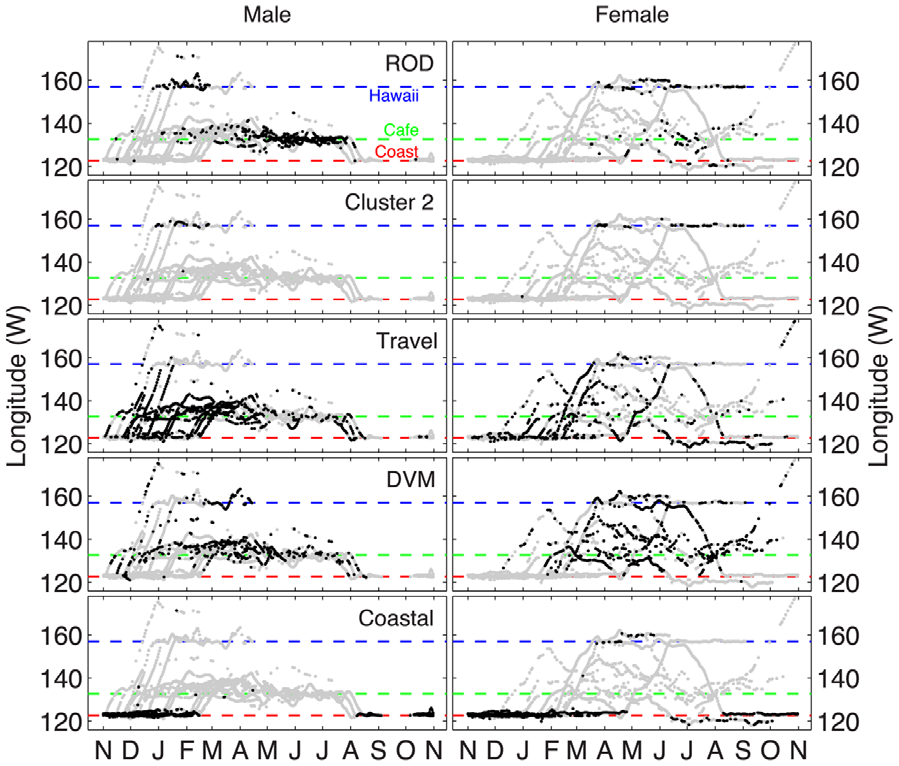
White shark seasonal and spatial patterns corresponding to each behavioral mode for males and females. The dotted lines represent the coast of California (red; near 122°W), the Café (green; near 135°W) and Hawaii (blue; near 156°W) respectively. All longitude estimates for the entire male (left panels) and female (right panels) dataset are shown in grey in the background with only the relevant data for each cluster and sex highlighted in black.

The white shark diving behavior clusters showed seasonal and sex related patterns (Figure 4). For both sexes the ‘Coastal’ mode occurred almost exclusively along the North American coast during fall and winter. Two females exhibited ‘Coastal’ behavior near Hawaii during spring. Diel vertical migration occurred among both males and females in virtually all offshore locations. However, among males DVM tapered off in spring as ROD became more dominant. ROD was largely a male diving behavior and occurred primarily in the Café between April and July.

**Figure 5:**
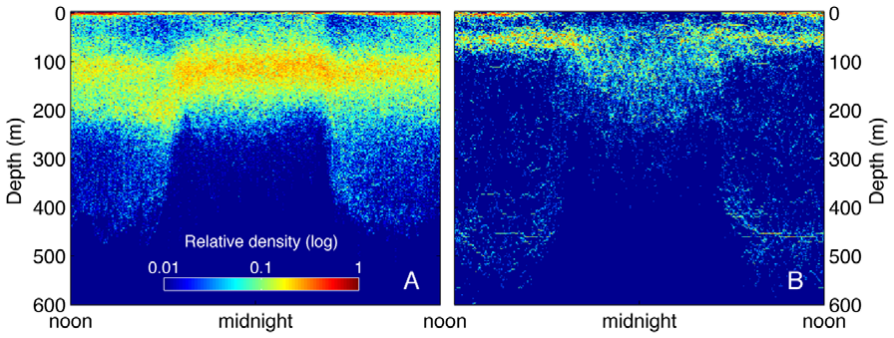
Differences in white shark behavior categorized as ‘ROD’ (rapid oscillatory diving) in the Café (a) and in Hawaii (b). High-resolution time and depth data were corrected for local time and aggregated by cluster over a 24-hour period. The relative density of data (log scale) is shown on a gridded surface. Clustering analysis placed most ROD in the Café, but some occurred in Hawaii. However, the high vertical swimming speed characteristic of ROD in the Café was not present in Hawaii. While the overall depth distribution was similar there were clear differences apparent at time-scales below the cluster data bin size (24 hrs) including a strong daytime density band around 50 m in Hawaii. These differences illustrate that the ROD behavior in the Café is unique.

There were key differences between Hawaii and the Café within the clustering assignment of ROD. Most ROD positions occurred in the Café, but some occurred in Hawaii for both males and females (Figure 4). Interestingly, these sparse Hawaii instances coincided closely in space and time with those from ‘cluster 2’ (see figure 4), a small cluster branching from the ROD group (see figure 1). Given this similarity, further comparisons between ‘cluster 2’ and ROD were noted. A density band in the 50–100 m bin characterized ‘cluster 2’, which differed from ROD (see Figure 1). When archival ROD data were segregated and compared between Hawaii and the Café (Figure 5), a 50 m density band occurred only in Hawaii, whereas the broader vertical distribution persisted in the Café. Furthermore, the mean vertical velocity for ROD was much greater in the Café (0.557 m/s) than in Hawaii (0.107 m/s) (Table 1; Wilcoxon test, p<0.05). These results indicate that despite some cross-categorization, ROD is a behavior largely unique to the Café. The distinction was not as discernable from clustering the 24-hour histograms, but was evident in the diel pattern and vertical velocity.

**Figure 6:**
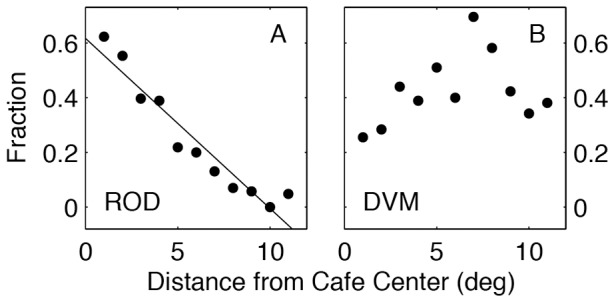
Spatial dependence of white shark ‘ROD’ (A) and ‘DVM’ (B) behavior in the Café. The regression shows that for ‘ROD’ the fraction of time (days) white sharks were engaged in ‘ROD’ declined steadily and linearly as a function of distance from the center of the Café region. In contrast no clear spatial relationship was evident for ‘DVM’.

ROD behavior was most concentrated toward the Café center. There was a strong negative linear relationship between ROD occurrence and distance from the center of the Café (R^2^ = 0.92, P<0.0001, F = 101.3). In contrast there was no obvious relationship between DVM and distance from the Café center (R^2^ = 0.12, P = 0.33, F = 1.1)(Figure 6). These results were consistent for males and females, although the relationship was strongest for males (Table 2).

**Table 2:** Results from linear regression of fraction of days white sharks engaged in diving behaviors over distance (degrees of longitude) from the center of the Café.

Cluster	R^2^	*F*	p-value
ROD_All_ *	0.9184	101.2930	0.0000
ROD_Male_	0.9167	99.0321	0.0000
ROD_Female_	0.6172	14.5121	0.0042
DVM_All_ *	0.1054	1.0607	0.3299
DVM_Male_	0.0000	0.0004	0.9839
DVM_Female_	0.3917	5.7950	0.0394

* _All_ refers to male, female and unknown sex.

There were distinct vertical velocity signatures associated with different diving modes and in some cases these differed by sex (Table 1). The Coastal mode was characterized by the lowest vertical velocity, and was similar for both males and females (∼0.04 m/s). Offshore, when not traveling, females moved on average at a consistent vertical rate (∼0.1 m/s) regardless of mode (DVM or ROD) or location (Hawaii or Café). In contrast, male movement was more rapid vertically for Travel, DVM and ROD. Mean vertical velocity was highest for ROD, particularly among males in the Café (0.57 m/s), where it was over 6 times higher than for females (see Table 1).

**Figure 7:**
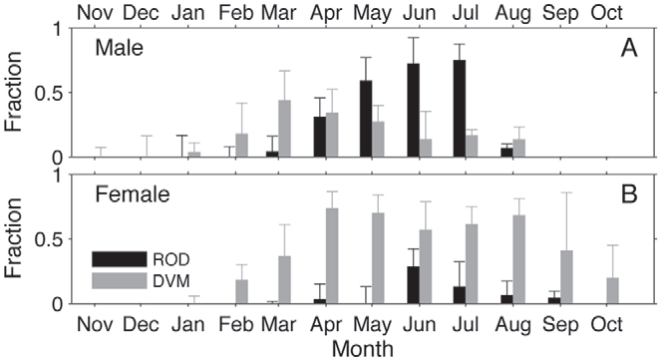
The fraction of time each month that male (A) and female (B) white sharks engaged in ‘ROD’ (black) and ‘DVM’ (grey) behavior. The bars represent median values, while the error bars show the upper inter-quartile range among individuals.

As white sharks first moved offshore in the winter (Figure 4) they engaged in DVM behavior (Figure 7). Gradually the occurrence of ROD became increasingly prevalent, and peaked in the Café between June through July for both males and females (Figure 7). During this period males spent a significantly greater fraction of time (median = 0.7241) than females (median = 0.0909) engaged in ROD (Wilcoxon test, p<0.0001). DVM remained the dominant mode for females during the entire offshore period.

## Discussion

The clustering analysis of time-at-depth data outlined here provides an effective method for distinguishing behavioral modes in telemetry records independent of *a priori* assumptions of how data might be grouped for comparison (i.e., by season or location). This method is generally applicable for archived and transmitted datasets provided data bins are consistent in their spatial-temporal resolution. Once behavioral clusters are distinguished, underlying patterns can be identified and linked to specific life history activities. In this instance we distinguished behavioral modes for white sharks based on vertical behavior from time-at-depth summary data.

The close match between behavioral clusters identified in this study and known behaviors described in previous telemetry studies provided a robust validation for the technique. The ‘Coastal’ cluster identified behavior consistent with *in situ* acoustic telemetry studies of white sharks ‘patrolling’ near seal rookeries [22], [26]. Goldman and Anderson [22] actively tracked individual white sharks over multiple days in the vicinity of the South East Farallon Island. They found that white sharks mostly swam near the substrate repeatedly patrolling the same areas and primarily occupying depths between five and 50 m. Cluster 5 in the present study (the ‘Coastal’ mode) similarly covered the same depth range (Figures 1 & 3), and virtually all days categorized in this cluster occurred along the west coast of North America. Klimley et al. [26] used an automated acoustic positioning system at Año Nuevo Island, and found no significant differences in day and night ‘patrolling’ behavior. Similarly, archival data from the ‘Coastal’ cluster indicated very consistent vertical patterning both day and night (Figure 3).

Further validation of the clustering results was evident for cluster 3, the ‘Travel’ behavioral mode. Previous studies have shown a preference for surface swimming with periodic ‘bounce’ dives during migratory movements by white sharks tagged in Central California, Guadalupe Island, South Africa, and New Zealand [4], [6], [7], [25]. This preference was shown clearly in the ‘Travel’ cluster that grouped days in which individuals swam almost exclusively at the surface with a faint trace of occasional deep dives (Figures 1 & 3).

Two dominant pelagic modes were clearly differentiated (DVM and ROD) when the sharks were not traveling. Our results show that white shark DVM occurs throughout their distribution in offshore northeastern Pacific waters. DVM has been linked to foraging in the deep scattering layer community in numerous marine predators including marine mammals [27], sea turtles [28], squid [29] and fishes [30], [31]. Isotopic studies confirmed white sharks forage while offshore [11] and we infer that white sharks are most likely foraging in the deep scattering layer when they exhibit the precise diel pattern distinguished in the DVM mode.

A compelling difference between the two dominant pelagic modes, DVM and ROD, was the spatial dependence of ROD. The occurrence of DVM throughout the white sharks offshore range is consistent with the link to foraging in the DSL. The DSL occurs broadly throughout the oceans, thus no strong spatial dependence for DVM diving behavior would be expected. In contrast, ROD occurred almost exclusively in the Café. Within the Café, this spatial dependence was further expressed as the frequency of ROD increased linearly (for males and females) with proximity to the center of the Café (Figure 6; Table 2) suggesting strong spatial targeting for ROD behavior.

Overall, these data demonstrate that, during spring, males converged and increasingly engaged in ROD towards the center of the Café. During the same period, a portion of females briefly visited the Café (13 of 15 still carrying PAT tags) and to a lesser extent also engaged in ROD increasingly toward the Café center (Table 2). Females occupied the same vertical distribution as males, but potentially moved at a much slower vertical velocity (Fig. 7; Table 1). Following the peak ROD season, males abruptly left the Café during August and returned to the North American coast. Shortly thereafter, females no longer engaged in ROD, yet many remained offshore longer, in some cases at least into October (Figures 4 & 7).

In evaluating the significance of the Café it is important to focus on its differentiating attributes. Our data show strong support that DVM is likely a foraging behavior, which occurred broadly across the entire white sharks pelagic range (including the Café). In contrast, ROD was characterized by a seasonal and geographic focus peaking at the Café center. This periodic focus and the associated increase in vertical swimming effort suggest a high value resource (food or other) that is seasonally available in a location consistent from year to year. Therefore, our discussion focuses specifically on whether ROD in the Café represents foraging or mating.

### Foraging hypothesis

The hypothesis that white sharks, particularly males, converge in the Café area during spring for foraging has been presented and discussed previously [6]–[8], [21], [32]. Rather than review this information, we present only additional discussion that adds a new perspective in light of the findings of our work.

If ROD is centered on a forage resource, it is clearly unlike the deep scattering layer community, which is likely targeted when white sharks exhibit DVM. Our results indicate that ROD and DVM vary in 1. diel pattern in depth distribution, 2. vertical velocity, 3. spatial distribution and 4. seasonality. The foraging hypothesis therefore predicts that individuals engaged in ROD are targeting a different prey source than those primarily engaged in DVM. These individuals should therefore have a different dietary composition compared with individuals engaged less in ROD (i.e., males should differentiate from females).

A further prediction of the foraging hypothesis is that the target resource should be seasonally concentrated toward the center of the Café where ROD was increasingly expressed. Potential prey resources such as marine meso-predators might be seasonally aggregated in the Café. For example, spawning squid [33] could be targeted either directly by white sharks, or could attract other predators, such as tunas [1] to aggregate and become the target of white shark foraging. Another potential concentrating mechanism is the shoaling of the O_2_ minimum layer adjacent to the Café, which could potentially concentrate prey through habitat compression [14].

Carlisle et al. [11] demonstrated through the stable isotope composition of muscle that male white sharks (most samples were male) indeed consumed prey during the offshore phase of migration. However, the isotopic ratios measured suggested that individuals foraged offshore at approximately half the rate they did during the coastal phase (the sample size for females was insufficient to draw any sex-specific difference). This result could be explained by the finding here that male white sharks engaged in DVM only a fraction of the time while offshore (Fig. 7). It could also mean that during ROD, males consumed prey that more closely reflected a coastal eastern Pacific signature (i.e. a migrant food source). However, the result could simply indicate that white sharks did not feed during ROD.

### Mating hypothesis

The mating hypothesis suggests that the primary resource of interest in the Café is the presence of mates, rather than food, and predicts that ROD is expressed when encountering or selecting mates. Our data indicate that as ROD activity increased during spring (Fig. 7), males converged to the more central region of the Café, whereas females occupied a much broader area and made only brief visits to the central Café area [8]. At first glance this partial segregation of sexes may be noted as evidence against the mating hypothesis [10], however, the pattern is in fact consistent with the mating systems of numerous migratory species, particularly those that form mating leks [34].

A lek is loosely defined as an aggregation of males that females visit with the specific purpose of finding a mate. Lekking is a purported mechanism for sexual selection, which may explain its broad prevalence and independent evolution across taxa. Among birds (which represent the majority of lek studies), lekking has evolved independently over 14 times and lek-like mating systems have been identified in insects, ungulates, bats, reptiles and fish [34]. Though such behavior is difficult to observe in fishes, lek-like mating has been shown in the families Synodontidae [35], Balistidae [36], Gadidae [37], Salmonidae [38], Cichlidae [39], Cyprinidae [40], Sparidae, Labridae, Scaridae, Acanthuridae, Poeciliidae, Characidae and Centrarchidae [41], and is thought to be even more likely in species that reproduce through internal fertilization [34].

The movement and behavioral patterns revealed in this study of white sharks are consistent with the general patterns and requirements of lek systems suggested by Bradbury [42]: 1) *no male paternal care*. There is no parental care in male white sharks. 2) *males are aggregated in an arena where females come solely for the purposes of mating*. The Café is a definable area where males seasonally aggregate in the Spring prior to female arrival [8], [10]. Female white sharks occasionally enter the Cafe, but remain for shorter periods of time and use a much larger area in general than males during these spring months. 3) *There is no unique resource to females except the males themselves*. If DVM is a proxy for foraging in the pelagic setting, its broad occurrence suggests a dispersed resource that is not unique to the Café. Thus females likely would not depend specifically on this central location for foraging. Finally, 4) *female mate choice*. In lek mating systems sexual selection can occur through female mate choice or endurance rivalry [43]. Mate choice requires females to make comparisons among males and select individuals with certain phenotypes [44]. In contrast, endurance rivalry is a mechanism of sexual selection where mating opportunities simply increase with time spent at the mating site [45]. Endurance rivalry should favor body condition or stores enabling costly mating activities over longer periods. For example, male grey seals, *Halichoerus grypus*, with greater body fat and energy are able to sustain mating for longer [46]. Female choice involves making comparisons among males, presumably through visual cues. This mechanism is challenged by the large spatial scale of the Café area and general low visibility in the ocean. However, the Café occurs in an oligotrophic region with the lowest surface chlorophyll in the northeastern Pacific, and therefore likely the greatest water clarity.

Alternatively, endurance rivalry could explain the rapid vertical movement of males during ROD. Precopulatory cues in elasmobranchs are thought to be principally olfactory, and reproductive receptiveness to copulation signaled by the release of female pheromones [47]. Diffusion in stratified water is far greater along isoclines than across, thus, a vertical search pattern would be most efficient in locating a horizontally dispersed scent plume. Extensive movement through the water column occurs during ROD. Males might be moving repeatedly across isoclines searching for traces of female pheromones, which they could then track to the source. In this scenario, those males that cover more area searching would be more likely to encounter females. However, maintaining ROD behavior for up to three months is probably energetically challenging, and may favor more fit individuals with greater endurance.

The location of the Café as a potential mating area also may be influenced by female white shark movement patterns. Our data suggest that females move more broadly and remain longer in the sub-tropical oceanic environment, particularly near Hawaii (see figure 4). This longer tenure of some mature females offshore has been cited as being linked to a potential biennial mode of reproduction [48], [49]. The selection of warmer water by females of some shark species may favor embryonic development [50]. The Café is located approximately midway between Hawaii and the coastal aggregation sites along the west coast of North America including the near-shore nursery grounds south of Point Conception [51]. Thus the Café location may be optimal in terms of potential costs associated with migration distance [9] for both sexes.

The hypothesis of lek-like mating in white sharks should be tested in future studies. The mating systems of oceanic sharks are poorly understood, however, similar sexual segregation has been noted in all other lamnid shark genera potentially indicating equivalent complex mating systems. Shortfin mako sharks (*Isurus oxyrinchus*) segregate by sex over comparable spatial scales in the open ocean [52]. Salmon sharks (*Lamna ditropis*) segregate during coastal periods but may overlap in the open ocean [53]. Klimley et al. [54] hypothesized that female hammerhead sharks (*S. lewini*) aggregate in segregated schools, and males compete for access to the largest females who favor the school center. Female aggregation may facilitate female choice and selection in mating systems of some elasmobranchs [47]. By contrast, if verified, the occurrence of sexual selection whereby male white sharks aggregate in the Café and individual females visit briefly for mating would be novel among shark species.

### Future hypothesis-driven studies

The ability to tease apart the intricate behaviors of migrating white sharks represents an important step forward in understanding their ecology. Still, the primary significance of the white shark Café, whether mating or foraging, cannot be conclusively determined from this study. However, the detailed knowledge gained here sets the stage for specific tests to directly evaluate the predictions generated. Future studies should strive to use tagging technologies and techniques that are increasingly hypothesis driven [55], [56] in combination with other disciplines such as stable isotope biochemistry, physiology, and genetic analysis.

Following the clear classification of distinct diving behavioral modes, stomach tags deployed via feeding [57], [58] could be used to test whether ROD is associated with foraging. Distinctive dips in stomach temperature associated with the ingestion of seawater along with prey items [59] could be measured during behavioral states classified as ROD from logged time-depth measurements. Additionally, since our data show that males engage preferentially in ROD over DVM, stable isotopes could be used to compare males and females and might confirm the hypothesis that ROD represents foraging on a specific prey targeted by males.

Miniaturized camera tags could also be deployed to capture images during ROD behavioral states. Such cameras have been successfully deployed over short time-scales on marine species including tiger sharks [60] to identify potential prey targets. A direct camera observation could confirm the encounter of prey versus mates during ROD. Another tagging technology that holds promise for this line of investigation is the ‘business card’ acoustic transmitter/receiver tag [61]. Each tagged white shark tag could carry a transmitter and logging receiver to test a mating hypothesis by quantifying the relative encounter rates of males and females in the Café during the peak spring period. Targeted applications of tagging technologies and analysis techniques, such as the clustering approach described here, provide a mechanism for future research with an increased potential for resolving the mechanisms underlying habitat use.

### Conclusion

Due to the over-reliance on a few recovered PAT tags with archival records, previous analyses have either suggested that ROD occurs in the Café and DVM occurs elsewhere, or failed to differentiate the two behaviors altogether and concluded that a single behavior, “foraging”, was prevalent in the Café [7], [21]. By utilizing vertical information from the entire transmitted dataset in this analysis, clear differences in behavior and seasonal timing between ROD and DVM became apparent. These new results provided an opportunity to further evaluate the two primary hypotheses on the importance of the Café region for northeastern Pacific white sharks and revealed previously unrecognized behavioral patterns resembling lek-mating systems in other organisms.

Though future efforts should focus on empirical evidence to definitively determine the specific biological actions and consequences represented by these clustered behaviors, the approach outlined here provides an objective analysis technique for distinguishing temporally and/or geographically disparate behaviors, an indication of life-history strategy, during periods when the subjects are otherwise not observable.

The application of clustering analysis on pooled summary data has provided a way to clearly distinguish between behaviors and generate statistical comparisons even where the behaviors overlap in space and time (in this case in the Café during spring). This methodology can be applied to other species with similar data types and has the potential to describe spatially and temporally specific behaviors, indicate sensitive or unique areas, as well as reveal general life-history strategies. This represents an important step forward for analyzing PAT tag data and determining behavior in otherwise unobservable phases.
